# An image-processing method to detect sub-optical features based on understanding noise in intensity measurements

**DOI:** 10.1007/s00249-017-1273-z

**Published:** 2018-02-01

**Authors:** Tripta Bhatia

**Affiliations:** 1 Raman Research Institute (RRI), Sir C. V. Raman Avenue, Bangalore, 560080 India; 2 Max Planck Institute of Colloids and Interfaces, Theory and Bio-Systems, 14424 Potsdam, Germany

**Keywords:** Image noise, Signal-to-noise ratio, Optimum smoothening, Tubules, Fluid lamellar phase

## Abstract

Accurate quantitative analysis of image data requires that we distinguish between fluorescence intensity (true signal) and the noise inherent to its measurements to the extent possible. We image multilamellar membrane tubes and beads that grow from defects in the fluid lamellar phase of the lipid 1,2-dioleoyl-*sn*-glycero-3-phosphocholine dissolved in water and water-glycerol mixtures by using fluorescence confocal polarizing microscope. We quantify image noise and determine the noise statistics. Understanding the nature of image noise also helps in optimizing image processing to detect sub-optical features, which would otherwise remain hidden. We use an image-processing technique “optimum smoothening” to improve the signal-to-noise ratio of features of interest without smearing their structural details. A high SNR renders desired positional accuracy with which it is possible to resolve features of interest with width below optical resolution. Using optimum smoothening, the smallest and the largest core diameter detected is of width $88 \pm 23$ and $6860 \pm 50$ nm, respectively, discussed in this paper. The image-processing and analysis techniques and the noise modeling discussed in this paper can be used for detailed morphological analysis of features down to sub-optical length scales that are obtained by any kind of fluorescence intensity imaging in the raster mode.

**Electronic supplementary material:**

The online version of this article (10.1007/s00249-017-1273-z) contains supplementary material, which is available to authorized users.

## Introduction

Membrane tubes are found in cells connecting different membrane compartments (Lee and Chen 1988). Tubes are suggested to play an important role in vesicular transport pathways (Simunovic et al. 2016) and ultrafast endocytosis pathways (Boucrot et al. 2015; Renard et al. 2015) in cells. In general, tubes can either be composed of a single lipid bilayer (unilamellar tubes) of thickness $\sim 5$ nm or of many (multi-lamellar tubes, MLTs). In vitro experiments performed using the synthetic membrane compartments such as free-standing giant unilamellar vesicles (GUVs) demonstrate that unilamellar tubes can form spontaneously if the two leaflets of the bilayer face different chemical environments (Simunovic et al. 2015; Li et al. 2011; Dosti et al. 2017) or have compositional differences (Lipowsky 2013) and can also be pulled in/out by applying local mechanical forces (Rossier et al. 2003).

**Fig. 1:**
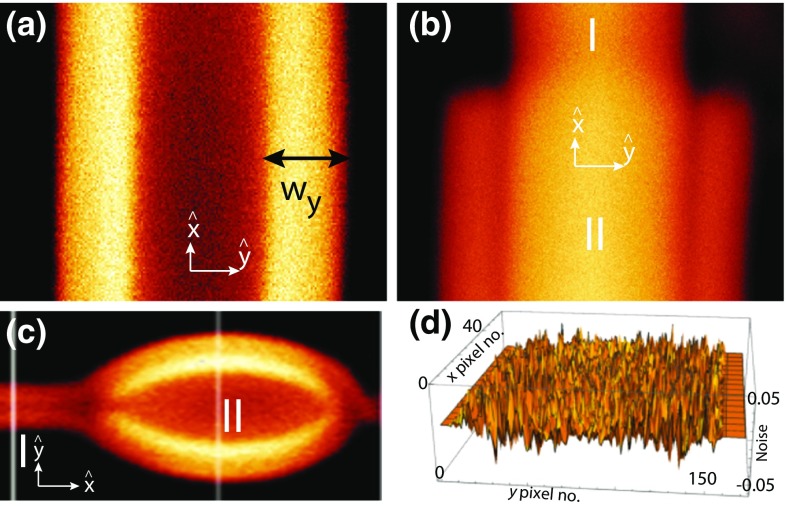
Fluorescence confocal polarizing microscope (FCPM) images of tubes where the incident laser beam is polarized along the long axis of tubes (*x*-axis). **a** A simple uniform tube with central water core and uniform core radius $r_{\mathrm {c}}$ and outer radius $r_{\mathrm {o}}$ along the length of the tube. The width of one of the bright band is shown by $w_{\mathrm {y}}$. **b**, **c** A coaxial tube and a bead on a tube with additional layers outside the main tube. I and II show the regions of the tube where number of lamella are different. The images shown here are taken at the widest horizontal cross section of the tubes. **d** Noise in the image for a small portion ($58 \times 170$) pixels of the image shown in **a**

In this paper, we have discussed membrane tubes (Bhatia et al. 2015) of the lipid, 1,2-dioleoyl-*sn*-glycero-3-phosphocholine (DOPC) that grow from the defects in the multilamellar lipid reservoir hydrated in excess water. Figure 1a shows a fluorescence confocal polarizing microscope (FCPM) image of a tube with a central core filled with solvent having uniform core and outer diameter along the long-axis. Figure 1b, c show FCPM image of tubes with shape asymmetry, i.e., size of tube is varying (at regions I and II) along the tube’s long-axis (*x*-axis). The bead shown in Fig. 1c is a prolate-ellipsoidal structure, which appears on some tubes. We have found that near the neck of the beads, the inner core of the tubes is not blocked by lipid material, i.e., the inner core runs continuously through the tubes and the beads. However, the core radii of the tube and the bead are different. We have taken multiple confocal *xyz* scans of the tubes and have found that growth (or retraction) of tubes is considerably slowed down for a couple of days after the sample cell is sealed and no further morphological shape changes occur as described in a previous paper (Bhatia et al. 2015). Upon sealing of the sample cell tubes do not add on to or give away the lipid material to and from the lipid reservoir, and hence can be considered as quasi-static and structurally stable objects in osmotic equilibrium. The confocal *xyz* scans for the tubes shown in Fig. 1a–c are shown in the Movie1.avi, Movie2.avi, and Movie3.avi, respectively, in the Supplementary Materials. Figure 1d shows noise spread in the region ($58 \times 170$) pixels of the image shown in Fig. 1a with a value between $[-0.06,0.05]$ that is extracted from the image, as described in the Results section.

In this paper, we describe image-analysis techniques that are used to reduce noise inherent in the intensity measurements. Reducing the noise without affecting the signal helps to detect the shape parameters of MLTs such as core diameter, outer diameter, and lamella thickness with an accuracy down to $\sim 23$ nm. The image-processing and analysis techniques and the noise modeling discussed in this paper can be used for any kind of fluorescence image data obtained in the raster mode and is not limited to confocal images.

## Methods

We have prepared lamellar stacks of the lipid DOPC (purchased from Sigma-Aldrich) onto a glass coverslip by spreading about 20–50 ${\upmu }$l of lipid solution in chloroform (containing about 0.2 mol% of the membrane dye, lissamine rhodamine B 1,2-dihexadecanoyl-*sn*-glycero-3-phosphoethanolamine, triethylammonium salt, RhPE purchased from Molecular Probes). The sample was gently dried under a nitrogen stream and kept covered inside a desiccator overnight with little vacuum sufficient to hold the chamber tight during the whole duration. A coated coverslip with dried sample was glued to a larger coverslip at the edges using mica spacers of about 100 $\upmu$m thickness. Solvent was introduced between the coverslips of sample cells by capillary action. The open edges were sealed using silicone glue immediately after the solvent filled the whole gap. We found that in the sealed sample cells, an osmotic equilibrium is reached between the swollen lamellar stack and excess water and the tubes remain stable for a couple of days. Sample cells are observed under a confocal microscope (LEICA TCS-SP2, He–Ne laser 543 nm) equipped with a 40$\times$ dry objective (0.85 N. A.) having a correction collar. Compared to a conventional microscope, the image contrast in the confocal microscope is improved by introducing two pinholes; (1) an excitation pinhole or illuminating aperture, and (2) an emission pinhole or confocal aperture. The first pinhole facilitates a point illumination of the tube by exciting fluorophores only in a small confocal volume defined by the axial ($\delta z$) and in-plane point spread function ($\delta x$, $\delta \mathrm{{y}}$) of the microscope optics. The second pinhole allows a fluorescence signal only from the confocal volume to reach the detectors (Pawley 2006). The detector converts light intensity into a corresponding quantity of electric charge carriers. Such charge carriers are generated by the signal of interest with non-zero mean [$I_\mathrm{{av}}(y)$] as well as due to random fluctuations in the signal intensity, namely noise with zero mean. Noise in general can be classified into three categories (Pawley 2006; Barlow 1999): (1) thermal noise, generated by system electronics, (2) Poisson noise, from random fluctuations in photon arrival time over a fixed period of time (given the mean) at the detector, and (3) dark noise, generated in the process of analog-to-digital conversion in the instrumentation and the random signal produced by photosensitive devices such as PMTs, photodiodes, or charge-coupled devices (CCDs) in the absence of any incident signal. A thin 2D planar optical-slice (or *xy* plane) of the tube is constructed by physically moving the focal spot with area ($\sim \delta x \delta \mathrm{{y}}$) from one edge of the sample to the other end with a chosen spatial sampling interval or scanning pixel width ($\Delta x$, $\Delta \mathrm{{y}}$) and fixed confocal *z*-slice thickness ($\delta z$). In order to build a 3D image, the confocal objective is moved by a controlled step in the *z*-direction to image the next consecutive 2D optical slice. Having all the 2D optical slices and knowing the consecutive z-steps, a computer program is used to build a 3D image of the sample. We have experimentally measured the point spread function (PSF) of the microscope optics ($\delta x$, $\delta y$, $\delta z$) for 40$\times$ objective by taking *xyt* and *xyz* scans of 200-nm isolated polystyrene fluorescent beads ($\lambda _\mathrm{{abs}}=488$ nm, $\lambda _\mathrm{{em}}=515$ nm) settled on the cover-slip in water (Sibarita 2005). We have analyzed ten fluorescence beads. From the Gaussian fit, we get $\delta x = \delta y = 0.56 \ \upmu$m and $\delta z = 2.56 \ \upmu$m.

### Positional accuracy

**Fig. 2:**
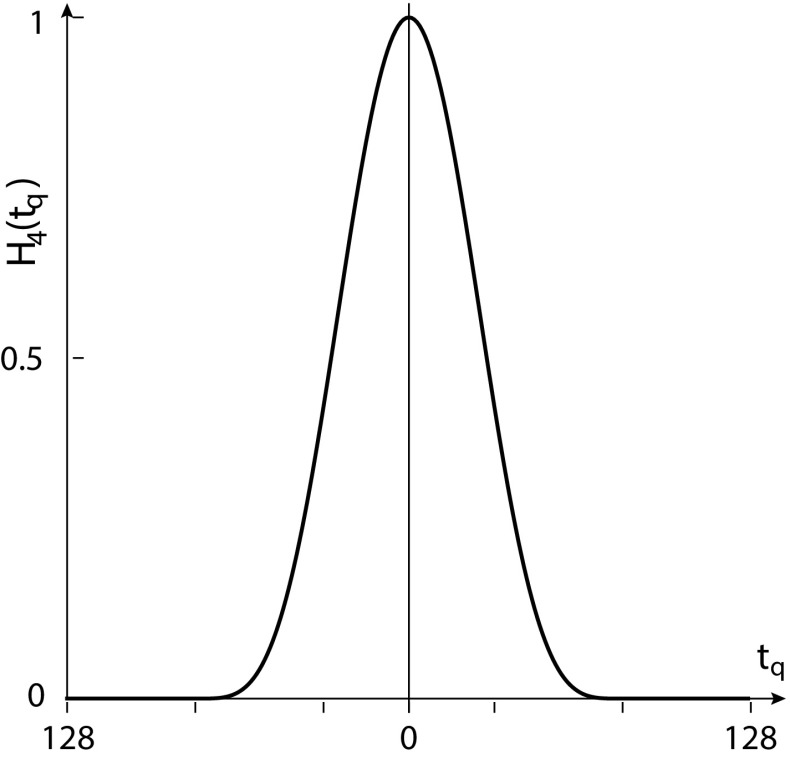
A 1-D low-pass filter, namely, double-Hann filter, $H_{4}(t_\mathrm{{q}})$ (*black*) is shown for $m_\mathrm{{q}}=160$ and $N_\mathrm{{y}}=256$ in Fourier domain where $t_\mathrm{{q}}$ is a discrete number of spectral points defined in the text

Rayleigh criterion considers only the limited numerical aperture of the objective to define resolution. Orhaug (1969), Falconi (1964), and Fried (1979) showed that the uncertainty in determining the position of a point source also depends on the signal-to-noise ratio (SNR $=I_{\mathrm {av}} / \sigma$) and the spatial sampling interval. The positional uncertainty is defined by Downs and Reichley (1983).

1 $$\begin{aligned} \frac{(\sigma /I_{\mathrm {av}}) }{\sqrt{\Big ( \sum _{k=1,. . n}(\Delta I_{k } / \Delta \mathrm{{y}}_{k }) \Big ) ^2}}, \end{aligned}$$

where $\sigma$ is the root mean square deviation of the noise and has the dimension of intensity, $I_{\mathrm {av}}$ is the average intensity, $I_{k }$ is the intensity at *k*th point, with *N* points in the profile and $\Delta \mathrm{{y}}_{k }$ is the spatial sampling interval at *k*th point. We have obtained confocal images of the narrow features with sampling interval finer than Nyquist sampling. The two length scales in the image have orders of magnitude difference that are associated with (1) the width $w_\mathrm{{y}}$ of the feature of interest and (2) the pixel width or sampling interval $\Delta \mathrm{{y}}$. Given that ($\Delta \mathrm{{y}} \ll w_\mathrm{{y}}$) , it is possible to separate the Fourier components that correspond to the true signal (confined to lower spatial frequencies) and the random fluctuations in the true signal present in each pixel (confined to higher spatial frequencies). For an image having $N_\mathrm{{y}}$ pixels (or data points) along *y*, if $\Delta y$ is the sampling width in the image domain, then corresponding sampling width in the Fourier domain is given by $\Delta {q_\mathrm{{y}}}=1/[N_\mathrm{{y}} \Delta {y}]$. We explore the possibility to improve the SNR of the feature based on the fact that the signal intensities are correlated over the length scale of the feature-width whereas random fluctuations in the observed intensities are not. We preferentially attenuate the noise in the higher spatial frequencies using suitable spatial frequency filter (low pass), amounting to smoothening in the image domain. We optimize the extent of smoothening operation for a particular feature of interest (e.g., one of the bright bands in the image) such that only noise is reduced without affecting the signal contribution. Let $y_{\mathrm {s}}$ be the scale of smoothening in the image domain that corresponds to $q_{\mathrm{{y}}_{\mathrm {s}}}$ in the Fourier domain. For an image having $N_\mathrm{{y}}$ pixels (or data points) along *y*, the scale of smoothening will be $m=(y_{\mathrm {s}}/\Delta \mathrm{{y}})$ pixels in the image domain corresponding to $m_\mathrm{{q}}=(N_\mathrm{{y}}/m)$ spectral points in the Fourier domain. The low-pass filter used to smoothen our images is the 1-d double-Hann filter in Fourier domain shown in Fig. 2. This filter response smoothly goes to 0 at $\left| q_\mathrm{{y}}\right| =q_{\mathrm{{y}}_{\mathrm {s}}}/2$ and beyond and is defined as

2 $$\begin{aligned} H_{4}(q_\mathrm{{y}})&= \cos ^{4} (\pi q_\mathrm{{y}} /q_{\mathrm{{y}}_{\mathrm {s}}}) , |q_\mathrm{{y}}|\le q_{\mathrm{{y}}_{\mathrm {s}}}/2 \nonumber \\&= 0, |q_\mathrm{{y}}|> q_{\mathrm{{y}}_{\mathrm {s}}}/2 \end{aligned}$$

For discretizing the window function, we express the Fourier variable $q_\mathrm{{y}} =(t_\mathrm{{q}} \ \Delta {q_\mathrm{{y}}})$ where $t_\mathrm{{q}}\in [-(N_\mathrm{{y}}/2) ,(N_\mathrm{{y}}/2) -1]$. The function becomes

3 $$\begin{aligned} H_{4}(t_\mathrm{{q}} \Delta {q_\mathrm{{q}}})&= \cos ^{4} (\pi t_\mathrm{{q}} /m_\mathrm{{q}}) , |t_\mathrm{{q}}|\le m_\mathrm{{q}}/2 \nonumber \\&= 0, |t_\mathrm{{q}}|>m_\mathrm{{q}}/2 \end{aligned}$$

## Results

**Fig. 3:**
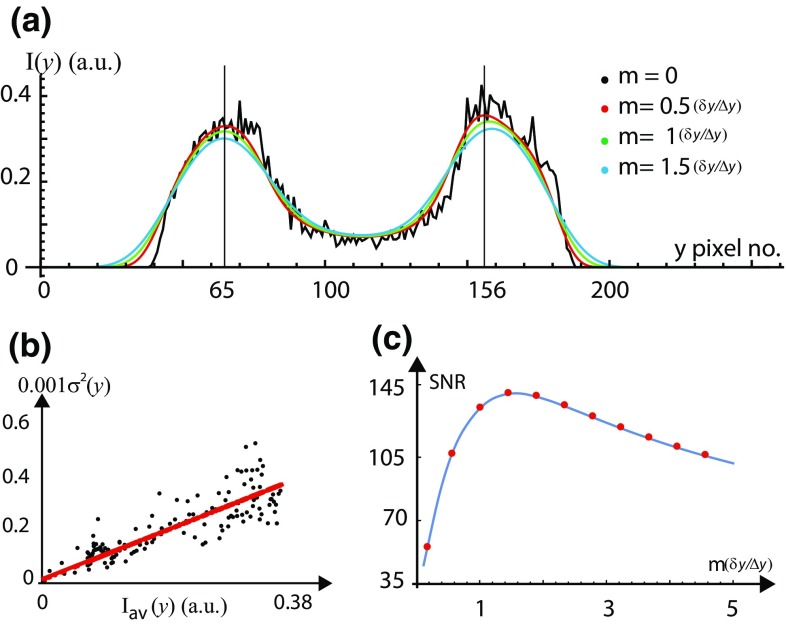
Improving the signal-to-noise ratio (SNR) of a feature in the image shown in Fig. 1a. **a** Transverse 1-D intensity profiles (arbitrary units, plotted against pixel number) for *m* = 0 (*black*), (0.5 $\delta _\mathrm{{y}}/\Delta \mathrm{{y}})$ (*red*), ($\delta _\mathrm{{y}}/\Delta \mathrm{{y}})$ (*green*) and (1.5 $\delta _\mathrm{{y}}/\Delta \mathrm{{y}})$ (*blue*) on top of each other for judging the smoothening operation. The PSF width in pixels is ($\delta _\mathrm{{y}}/\Delta \mathrm{{y}}\sim 12$) for this image. The intensity is maximum at pixel number $y=65$ and $y=156$ for the tube. **b** Plot of noise variance [$\sigma ^{2} (y)$] vs. average $1-$D fluorescence intensity [$I_{\mathrm{{av}}}(y)$] of the tube. The* red line* shows a model fit to the data. c Signal-to-noise ratio (SNR) is plotted as a function of the smoothening radius *m* in the units of $\sim (\delta _\mathrm{{y}}/\Delta \mathrm{{y}})$. The peak SNR is improved from $(I_{\mathrm{{av}}}(y) /\sigma (y) ) \simeq 53$ up to 145 by smoothening operation

The average intensity profile for tubes shown in Fig. 1 is given by $I_\mathrm{{av}}(y) = \sum _{k=1}^{N_\mathrm{{f}}} \left[ \sum _{j=1}^{N_{x}} I^{\mathrm{{f}}}(x_{j},y) /N_{x}\right] N_\mathrm{{f}}$, where $N_{x}$ is the number of rows averaged along *x* and $N_\mathrm{{f}}$ is the number of frames. The intensity deviation from the mean at a pixel location (*x*, *y*) in a frame, $I_{\mathrm {N}}(x,y) =[I(x,y) -I_\mathrm{{av}}(y) ]$ gives the noise distribution across the image. Figure 3a shows the smoothened intensity profiles for *m* = (0.5 $\delta _\mathrm{{y}}/\Delta \mathrm{{y}})$, ($\delta _\mathrm{{y}}/\Delta \mathrm{{y}})$ and (1.5 $\delta _\mathrm{{y}}/\Delta \mathrm{{y}})$ on top of the raw intensity profile for the tube. We restrict our discussion to 1-D variation of the fluorescence intensity along *y* at fixed *x*. We estimate the variance of the fluctuations in fluorescence intensity, $\sigma ^2 (y) = \sum _{k=1}^{N_\mathrm{{f}}}\sum _{j=1}^{N_{x}} [I_{\mathrm {N}}(x_{j},y) ]^2/N_{x}N_\mathrm{{f}}$ in the image. The red line in Fig. 3b shows the best model fit of the data (red color) with $\sigma ^2 (y) =10.10^{-4} \ I_\mathrm{{av}}(y) + 7.10^{-6}$. The statistical distribution of noise contaminating the intensity measurement is completely defined by the variance [$\sigma ^2 (y)$] and the mean [$I_{\mathrm{{av}}}(y)$]. We have (1) thermal noise for which $\sigma ^2_{\mathrm{{th}}}$ is proportional to the observed mean square fluorescence intensity $I_{\mathrm{{av}}}^2 (y)$, (2) Poisson noise for which $\sigma ^2_{\mathrm{{P}}} (y)$ is proportional to the observed mean fluorescence intensity $I_{\mathrm{{av}}} (y)$ and (3) dark noise for which $\sigma ^2_{\mathrm{{d}}} (y)$ is independent of the signal intensity. From the fit shown in Fig. 3b we have $\sigma ^2_{\mathrm{{d}}}=7.10^{-6}$ and $a_{\mathrm{{p}}}=[\sigma ^2_{\mathrm{{P}}}/I_{\mathrm{{av}}} (y) ]=10^{-3}$.

The SNR is defined as [$I_{\mathrm{{av}}}(y) /\sigma (y)$]. One of the bright bands in the image shown in Fig. 1a has a width $w_\mathrm{{y}}\simeq 1.65 ~\upmu \mathrm {m}$ with peak mean intensity $I_{\mathrm{{av}}}(y) \simeq 0.37$ at pixel number $y=156$ shown in Fig. 3a and $\sigma (y) \simeq 0.007$ [estimated using the relation between $\sigma ^2 (y)$ and $I_{\mathrm{{av}}}(y)$]. Thus, the peak SNR of this particular feature is $(I_{\mathrm{{av}}}(y) /\sigma (y) ) \simeq 53$. Noise can be reduced if and only if [$\sigma (y)$] is decreased without affecting the $[(I_{\mathrm{{av}}}(y)$] giving a higher SNR. However, if [$I_{\mathrm{{av}}}(y)$] is also decreased (by over-smoothening) together with [$\sigma (y)$] then this eventually results in a decrease in SNR. To know precisely the value of the radius of smoothening (*m*) at which we do over-smoothening, we have scrutinized the fluorescence intensity profiles for different values of *m* on top of each other with the raw intensity profile ($m=0$) shown in Fig. 3a. To assess an optimum smoothening scale, we systematically vary trial values of the scale of the smoothening *m* and examine the resultant SNR shown in Fig. 3c for $m \in [0.5(\delta _\mathrm{{y}}/\Delta _\mathrm{{y}}) ,5(\delta _\mathrm{{y}}/\Delta _\mathrm{{y}})]$, where the PSF width in pixels is ($\delta _\mathrm{{y}}/\Delta \mathrm{{y}}\sim 12$) for this image. As the radius of smoothening *m* is changed, the peak SNR of the feature is increased by 3.2 times from 53 up to 145 by smoothening operation. The magnitude of the SNR initially increases as we increase *m*, reaches a maximum at $m=1.5(\delta _\mathrm{{y}}/\Delta _\mathrm{{y}})$ and then reduces. From Fig. 3a, we find that the peak intensity of the selected feature (at pixel number *y* = 156 shown in Fig. 3a) starts to come down for $m>0.5(\delta _\mathrm{{y}}/\Delta _\mathrm{{y}})$, and hence $m=0.5(\delta _\mathrm{{y}}/\Delta _\mathrm{{y}})$ would be considered optimum for this feature, with SNR $\simeq 105$. Therefore, to retain the feature shape as intact as possible, we choose conservative smoothening, at the expense of (i.e., with less) SNR.

**Fig. 4:**
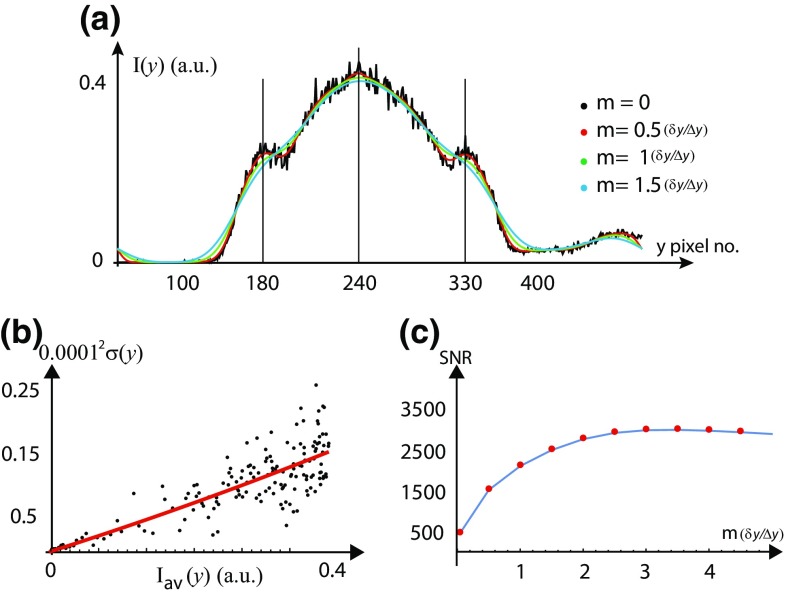
Improving the signal-to-noise ratio (SNR) of a feature in the image shown in Fig. 1b at cross section I. **a** Transverse 1-D fluorescence intensity profiles (arbitrary units, plotted against pixel number) are plotted on top of each other for $m=0$ (*black*), (0.5 $\delta _\mathrm{{y}}/\Delta \mathrm{{y}})$ (*red*), ($\delta _\mathrm{{y}}/\Delta y)$ (*green*) and (1.5 $\delta _\mathrm{{y}}/\Delta \mathrm{{y}})$ (*blue*), for judging the smoothening operation. The PSF width in pixels is ($\delta _\mathrm{{y}}/\Delta \mathrm{{y}}\sim 25$) for this image. The jump in the intensity profile beyond pixel no. $y=400$ is an artifact. **b** Plot of noise variance [$\sigma ^{2} (y)$] vs. average 1-D fluorescence intensity [($I_{\mathrm{{av}}}(y)$] of the tube. The* red line* shows a model fit to the data. **c** signal-to-noise ratio (SNR) is plotted as a function of the smoothening radius *m* in the units of $\sim (\delta _\mathrm{{y}}/\Delta \mathrm{{y}})$. The peak signal-to-noise ratio (SNR) of the feature is improved by six times from $(I_{\mathrm{{av}}}(y) /\sigma (y) ) \simeq 500$ up to 3000 by smoothening operation

For the tube shown in Fig. 1b, we have performed the smoothening operation for the feature shown at cross section I and the result of smoothening operation is shown in Fig. 4. We compare the raw intensity profiles of the tube with the smoothened intensity profiles shown in the Fig. 4a for $m=0$, (0.5 $\delta _\mathrm{{y}}/\Delta \mathrm{{y}}$), ($\delta _\mathrm{{y}}/\Delta \mathrm{{y}}$) and (1.5 $\delta _\mathrm{{y}}/\Delta \mathrm{{y}}$) where the PSF width in pixels is ($\delta _\mathrm{{y}}/\Delta \mathrm{{y}}\sim 25$) for this image. The peak mean intensity is $I_{\mathrm{{av}}}(y) \simeq 0.37$ at pixel number $y=240$ shown in Fig. 4a. We estimate the variance of the fluctuations in fluorescence intensity in the image and the red line in Fig. 4b show the best model fit of the data with $\sigma ^2 (y) =2.10^{-4} \ I_{\mathrm{{av}}}^2 (y) + 5.10^{-4} \ I_{\mathrm{{av}}}(y) + 3.10^{-7}$. From the fit, we have $\sigma ^2_{\mathrm{{d}}}=3.10^{-7}$, $a_{\mathrm{{p}}}=[\sigma ^2_{\mathrm{{P}}}/I_{\mathrm{{av}}} (y) ]=5.10^{-4}$ and $a_{\mathrm{{th}}}=[\sigma ^2_{\mathrm{{th}}}/I_{\mathrm{{av}}}^2 (y) ]=2.10^{-4}$. We estimate the $\sigma (y)$ using the relation between $\sigma ^2 (y)$ and $I_{\mathrm{{av}}}(y)$ and this gives the peak SNR of this particular feature as $(I_{\mathrm{{av}}}(y) /\sigma (y) ) \simeq 500$. As shown in Fig. 4c, if we increase the smoothening radius (*m*) , the SNR initially increases, and reaches a maximum at $m=3(\delta _\mathrm{{y}}/\Delta \mathrm{{y}})$ and then starts to reduce slowly. We have improved the peak signal-to-noise ratio (SNR) of the feature by six times from $(I_{\mathrm{{av}}}(y) /\sigma (y) ) \simeq 500$ up to 3000 by smoothening operation. From Fig. 4a we find that the peak intensity at pixel number $y=240$ and the knee intensity at pixel number $y=180$ and $y=330$ of the selected feature starts to come down for $m>0.5(\delta _\mathrm{{y}}/\Delta \mathrm{{y}})$. Hence, $m=0.5(\delta _\mathrm{{y}}/\Delta \mathrm{{y}})$ would be considered optimum for this feature, with SNR $\simeq 1600$.

**Fig. 5:**
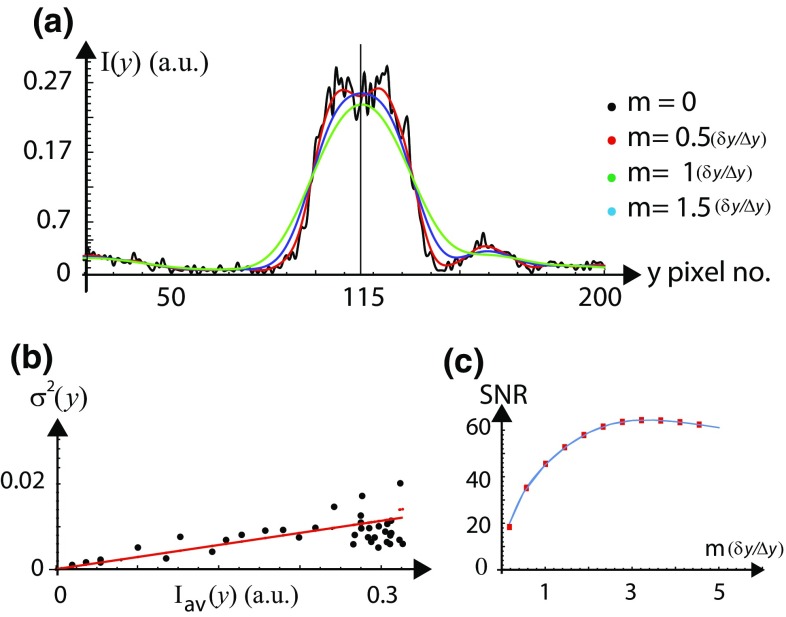
Improving the signal-to-noise ratio (SNR) of a feature in the image shown in Fig. 1c at cross section I. **a** Transverse 1-D fluorescence intensity profiles (arbitrary units, plotted against pixel number) are plotted on top of each other for $m=0$ (*black*), (0.5 $\delta _\mathrm{{y}}/\Delta \mathrm{{y}})$ (*red*), ($\delta _\mathrm{{y}}/\Delta y)$ (*green*) and (1.5 $\delta _\mathrm{{y}}/\Delta \mathrm{{y}})$ (*blue*), for judging the smoothening operation. The PSF width in pixels is ($\delta _\mathrm{{y}}/\Delta \mathrm{{y}}\sim 12$) for this image. The jump in the intensity profile before pixel no. $y=50$ and beyond pixel no. $y=140$ is an artifact. **b** Plot of noise variance ($\sigma ^{2} (y)$) vs. average 1-D fluorescence intensity ($I_{\mathrm{{av}}}(y)$) of the tube. The red line shows a model fit to the data. **c** Signal-to-noise ratio (SNR) is plotted as a function of the smoothening radius *m* in the units of $\sim (\delta _\mathrm{{y}}/\Delta \mathrm{{y}})$. The peak signal-to-noise ratio (SNR) of the feature is improved from $(I_{\mathrm{{av}}}(y) /\sigma (y) ) \simeq 20$ up to 64 by smoothening operation

For the tube shown in Fig. 1c, we have performed the smoothening operation for the feature shown at cross section I and the result of smoothening operation is shown in Fig. 5. We compare the raw intensity profiles of the tube with the smoothened intensity profiles shown in the Fig. 5a for $m=0$ (black), (0.5 $\delta _\mathrm{{y}}/\Delta \mathrm{{y}})$ (red), ($\delta _\mathrm{{y}}/\Delta \mathrm{{y}})$ (green) and (1.5 $\delta _\mathrm{{y}}/\Delta \mathrm{{y}})$ (blue) where the PSF width in pixels is ($\delta _\mathrm{{y}}/\Delta \mathrm{{y}}\sim 12$) for this image. The peak mean intensity is $I_{\mathrm{{av}}}(y) \simeq 0.37$ at pixel number $y=115$ shown in Fig. 5a. We estimate the variance of the fluctuations in fluorescence intensity in the image and the red line in Fig. 5b show the best model fit of the data with $\sigma ^2 (y) = 9.10^{-5} \ I_{\mathrm{{av}}}^2 (y) + 14.10^{-3} \ I_{\mathrm{{av}}}(y) + 11.10^{-5}$. From the fit, we have $\sigma ^2_{\mathrm{{d}}}=11.10^{-5}$, $a_{\mathrm{{p}}}=[\sigma ^2_{\mathrm{{P}}}/I_{\mathrm{{av}}} (y) ]=14.10^{-3}$ and $a_{\mathrm{{th}}}=[\sigma ^2_{\mathrm{{th}}}/I_{\mathrm{{av}}}^2 (y) ]=9.10^{-5}$. We estimate the $\sigma (y)$ using the relation between $\sigma ^2 (y)$ and $I_{\mathrm{{av}}}(y)$ and this gives the peak SNR of this particular feature as $(I_{\mathrm{{av}}}(y) /\sigma (y) ) \simeq 20$. As shown in Fig. 5c, if we increase the smoothening radius (*m*) , the SNR initially increases, reaches a maximum at $m=3.5(\delta _\mathrm{{y}}/\Delta \mathrm{{y}})$ and then starts to reduce slowly. We have improved the peak SNR of the feature from $(I_{\mathrm{{av}}}(y) /\sigma (y) ) \simeq 20$ up to 64 by smoothening operation. From Fig. 5a, we find that the peak intensity at $y=115$ of the selected feature starts to come down for $m>0.5(\delta _\mathrm{{y}}/\Delta \mathrm{{y}})$. Hence $m=0.5(\delta _\mathrm{{y}}/\Delta \mathrm{{y}})$ would be considered optimum for this feature, with SNR $\simeq 35$.

**Fig. 6:**
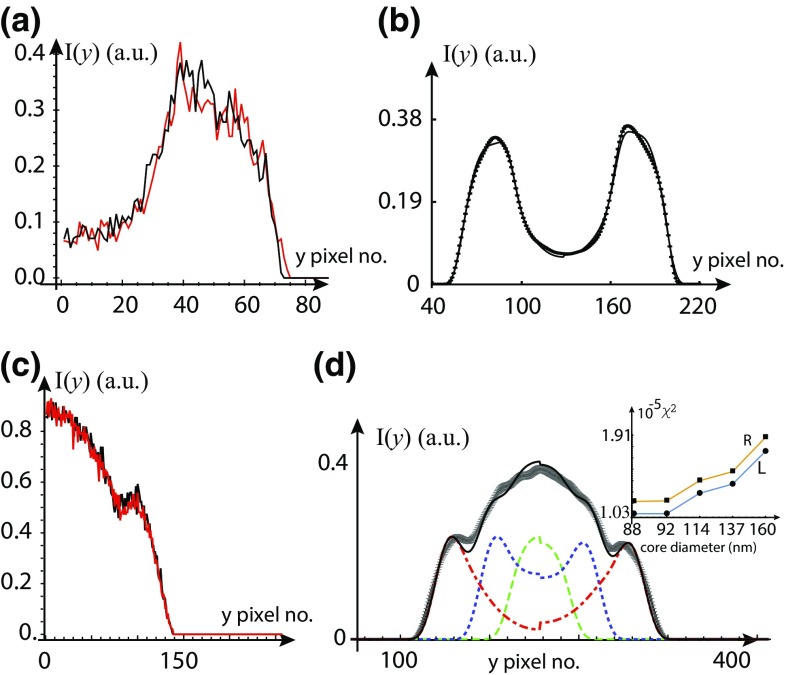
Finding symmetry axis and shape parameters of tubes. **a** The left and right halves of the tube are plotted on top of each other for a fixed *x* along *y*-axis. **b** Transverse 1-D experimental smoothened intensity profiles $I_{\mathrm {sm}}$ for $m=0.5(\delta _\mathrm{{y}}/\Delta \mathrm{{y}})$ (*black with error bars*) of the tube (shown in Fig. 1a) is plotted with the model intensity profile (*black*). From the fit, we get $r_{\mathrm {c}} = 1.74 \ \upmu \mathrm{{m}}$, $r_{\mathrm {o}} = 3.45 \ \upmu \mathrm{{m}}$ and $\alpha =0.35$. **c** For the tube shown in Fig. 1b at cross section II: the left and right halves of the tube are plotted on top of each other for a fixed *x* along *y*-axis. **d** Transverse 1-D experimental smoothened intensity profiles $I_{\mathrm {sm}}$ for $m=0.5(\delta _\mathrm{{y}}/\Delta \mathrm{{y}})$ (*black with error bars*) of the tube and model (*black curve*), constituent tubes (*dotted and dashed curves*) with $((r_{\mathrm {c1}},r_{\mathrm {o1}})$, $(r_{\mathrm {c2}},r_{\mathrm {o2}})$, $(r_{\mathrm {c3}},r_{\mathrm {o3}}) )$ = ((0.044, 0.92), (1.03, 1.69), $(2.17, 2.86) ) \ \upmu \mathrm{{m}}$, $\alpha =0.5$. In the inset, we plot the $\chi ^2$ values for the different value of the innermost core diameter (2r_c1_) for the left (L) and right (R) halves of the tube

## Advantage of optimum smoothening in the model fitting

We retain the images with their original sampling, which offers advantage at the model fitting stage as described below. For a given cross section of tube at *x*, the best fits are obtained by minimizing the weighted $\chi ^{2}(\alpha , r_{\mathrm {c}}, r_{\mathrm {o}}, \delta z)$ given by Bevington and Robinson (2002)

4 $$\begin{aligned} \chi ^{2} = \frac{1}{N} \sum _{j = 1}^{N} \, \left[ (\, I_{\mathrm {sm}}(y_{j}) - \alpha \sum _{k} I_{\mathrm {M}}^{(k) } (y_{j}; r_{\mathrm {c}}, r_{\mathrm {o}}, \delta z) \, ) / \sigma _{j} \right] ^{2}, \end{aligned}$$

where $I_{\mathrm {sm}}$ is the smoothened intensity profile chosen optimally, $I_{\mathrm {M}}$ is the model intensity profile, $\alpha$ is a scaling parameter required for the fit of $I_{\mathrm {sm}}$ with $I_{\mathrm {M}}$, $\sigma _{j}$ is the standard deviation of the noise associated with $j^{\mathrm {th}}$ pixel along the *y*-axis for a given *x* (calculated from noise modeling), and *k* labels separate concentric tubes in a given cross section. The number of pixels (*j*) are kept the same as originally chosen for image scanning, even though the intensity profile is smoothened with optimally chosen spatial smoothening scale. The model for the tubes to obtain the $I_{\mathrm {M}}$ in Eq. (4) is discussed in detail previously (Bhatia et al. 2015). We model the two halves (divided by the *xz* plane) separately to estimate the fitting parameters $(r_{\mathrm {c}}, r_{\mathrm {o}}, \delta z)$ because for asymmetric tubes (shown in Fig. 1b), the axis of shape asymmetry need not be same as the central axis of the tube.

We first consider the simple tube Fig. 1a. The folded left and right halves of the tube are plotted on top of each other, as shown in Fig. 6a, after finding the symmetry axis by cross correlation. We find that the tube consists of a single core. The best fit of the smoothened intensity profile for $m=0.5(\delta _\mathrm{{y}}/\Delta \mathrm{{y}})$ with the model intensity profile is shown in Fig. 6b. The error bars on the smoothened intensity profile are calculated from noise statistics [$\sigma ^2 (y) ,\ I_{\mathrm{{av}}}(y)$] at each pixel. From the best fit, we find: $(r_{\mathrm {c}},r_{\mathrm {o}})$ = $(1.74, 3.45) \ \upmu \mathrm{{m}}$ and $\alpha =0.35$.

Figure 6c shows the folded left and right halves of the cross section labeled II of the tube shown in Fig. 1b. For the best fit of the smoothened intensity profile for $m=0.5(\delta _\mathrm{{y}}/\Delta \mathrm{{y}})$ with the model intensity profile, this region requires three values of *k*, i.e., three separate tubes where the radii of various regions are denoted by ${r_{1}}$, ${r_{2}}$, ${r_{3}}$etc., as described in Eq. (4). From the best fit, we find that the cross section II consists of three tubes with shape parameters: $((r_{\mathrm {c1}},r_{\mathrm {o1}})$, $(r_{\mathrm {c2}},r_{\mathrm {o2}})$, $(r_{\mathrm {c3}},r_{\mathrm {o3}}) )$ = ((0.044, 0.92), (1.03, 1.69), $(2.17, 2.86) ) \ \upmu \mathrm{{m}}$, and $\alpha =0.5$. We plot the $\chi ^2$ values of the fit for the core diameter (2r_c1_) shown in Fig. 6d, where we vary the innermost core size from 0.046 to 0.16 ${\upmu {\mathrm {m}}}$ in the steps of 4–23 nm shown in the inset of Fig. 6d. We find that the values for $\chi ^2 (0.088\,\upmu {\mathrm {m}}) < \chi ^2 (0.092\,\upmu {\mathrm {m}})$ or for higher core size and $\chi ^2 (0.088\,\upmu {\mathrm {m}}) = \chi ^2 (0.084\,\upmu {\mathrm {m}})$ or for lower core size up to 46 nm (two pixels). The innermost core size, as decided by the minimum $\chi ^2$, is (46–88) nm with a sensitivity of ± 23 nm (one pixel) for the image shown in Fig. 1b. It is important to emphasize that it is possible to extract core sizes as small as 46–88 nm with a sensitivity of ± 23 nm (one pixel) as a result of optimum smoothening (shown in Fig. 4a) for $m=0.5(\delta _\mathrm{{y}}/\Delta \mathrm{{y}})$, which would otherwise remain hidden under the noise. For the tube shown in Fig. 1c, the shape parameters are provided in a previous paper (Bhatia et al. 2015).

## Conclusions

The image-processing, analysis techniques and the noise modeling discussed in this paper can be used for extracting the structural parameters of objects with features width at sub-optical and at or above optical length scales. The procedure is demonstrated by three different examples of tubes, as shown in Fig. 1. The image-processing procedure, “optimum smoothening”, has the potential to reduce the noise inherent to the intensity measurements during image formation without affecting the true signal. The SNR is defined as [$I_{\mathrm{{av}}}(y) /\sigma (y)$]. Noise is reduced if and only if [$\sigma (y)$] is decreased without affecting the [$I_{\mathrm{{av}}}(y)$] giving a higher SNR, as shown in Figs. 3c, 4c, and 5c for $m=0.5(\delta _\mathrm{{y}}/\Delta \mathrm{{y}})$. However, if data is over-smoothened, then [$I_{\mathrm{{av}}}(y)$] is also decreased together with [$\sigma (y)$] and this eventually results in a decrease in SNR. To know precisely the optimum value of the radius of smoothening (*m*) beyond which we start to lose [$I_{\mathrm{{av}}}(y)]$, we have scrutinized the fluorescence intensity profiles for different values of *m* on top of each other with the raw intensity profile ($m=0$) shown in Figs. 3a, 4a, and 5a. As the radius of smoothening, *m*, is changed in the half-integral values of the PSF width from 0.5 to 1.5, the value of the peak intensity at pixel number $y=156$ (in Fig. 3a) starts to come down for $m>0.5(\delta _\mathrm{{y}}/\Delta \mathrm{{y}})$. Similar behavior is seen in Fig. 4a at $y=180$ (knee intensity), $y=240$ (peak intensity) and 330 (knee intensity) and in Fig. 5a at $y=115$ (peak intensity). We have chosen an optimum value of *m* such that only random fluctuations are smoothened (noise is reduced) without affecting the peak or the knee intensity (true signal) shown in Figs. 3a, 4a, and 5a.

We have shown that optimum smoothening can be used to increase SNR. This helps in detecting the innermost core size of 46–88 nm with a sensitivity of ± 23 nm (one pixel), which would otherwise remain hidden in the noise. The only requirement for the imaging method is that it should be in the raster mode (pixel by pixel). The choice can be either standard confocal or super-resolution microscopes depending on the samples. In both cases, as long as we have separation of length scales in the images with feature width much larger than the sampling interval, it is possible to separate out the Fourier components of the noise and the true signal. The random noise associated with each pixel cannot be avoided but can be minimized by using a low-pass filter, such as a 1-D double Hann filter. The choice of low-pass filter may vary from sample to sample depending on the nature and dynamics of the feature of interest. We choose the 1-D double Hann filter because in the image domain, the associated smoothening function of $H_{4}(t_\mathrm{{q}})$ has lower side-lobe levels. This reduces the possible “ringing” effect in the image. The method described here can be used to extract shape parameters of features such as membrane tubes (or beads) that are reported previously (Simunovic et al. 2015, 2016; Boucrot et al. 2015; Renard et al. 2015; Domanov and Kinnunen 2006; Domingues and Miranda 2010; Arouni et al. 2011; Stossel and Hartwig 2006; Virchow 1854; Chapman and Fluck 1966; Sandermann and Vatter 1977; Sakurai and Sakurai 1989; Zou and Nagel 2006; Zou 2009; Huang et al. 2005). The choice of membrane dye is arbitrary because it is possible to find out independently the orientation of the dye in the lipid bilayer to model the intensity profiles.

With oversampling in our images at sampling frequency much larger than the Fourier components corresponding to the signal, we have explored the possibility of optimum smoothening in the image domain. Finding the statistics of the image noise, i.e., the relation between $\sigma ^2 (y)$ and $I_{\mathrm{av}}(y)$ is helpful to model the noise, which is used to extract and reduce the random fluctuations in the signal intensity at each pixel in the image. By understanding the nature of the noise, we try to optimize the image processing by optimizing the scale of smoothening in order to increase the SNR for a feature of interest. The results from application of this method are used for further modeling the structure of the tubes and beads of a common well-hydrated phospholipid DOPC in the $L_{\alpha }$ phase. We find that almost all tubes have a core, with a few tubes having a core diameter below the optical resolving limit. We could detect features of size as small as $(88\pm 23)$ nm by enhancing the SNR using optimum smoothening without deconvolution.

## Electronic supplementary material

Below is the link to the electronic supplementary material.

Supplementary material 1 (AVI 106 KB)

Supplementary material 2 (AVI 215 KB)

Supplementary material 3 (AVI 94 KB)
